# The Urinary Glycopeptide Profile Differentiates Early Cardiorenal Risk in Subjects Not Meeting Criteria for Chronic Kidney Disease

**DOI:** 10.3390/ijms25137005

**Published:** 2024-06-26

**Authors:** Aranzazu Santiago-Hernandez, Marta Martin-Lorenzo, María Gómez-Serrano, Juan Antonio Lopez, Ariadna Martin-Blazquez, Perceval Vellosillo, Pablo Minguez, Paula J. Martinez, Jesús Vázquez, Gema Ruiz-Hurtado, Maria G. Barderas, Pantelis Sarafidis, Julian Segura, Luis M. Ruilope, Gloria Alvarez-Llamas

**Affiliations:** 1 Immunology Department, Instituto de Investigación Sanitaria Fundación Jiménez Díaz-UAM, 28040 Madrid, Spain; aranzazu_sant@hotmail.com (A.S.-H.); marta.martin@fjd.es (M.M.-L.); ariadna.martinb@quironsalud.es (A.M.-B.); paula_jmg@hotmail.com (P.J.M.); 2 Fundación Jiménez Díaz University Hospital-UAM, 28040 Madrid, Spain; perceval.vellosillo@gmail.com (P.V.); pablo.minguez@quironsalud.es (P.M.); 3 Laboratory of Cardiovascular Proteomics, Centro Nacional de Investigaciones Cardiovasculares, 28029 Madrid, Spain; maria.gomezserrano@imt.uni-marburg.de (M.G.-S.); jalopez@cnic.es (J.A.L.); jesus.vazquez@cnic.es (J.V.); 4 Center for Tumor Biology and Immunology (ZTI), Philipps University, 35043 Marburg, Germany; 5 CIBER de Enfermedades Cardiovasculares (CIBERCV), 28041 Madrid, Spain; gemaruiz@h12o.es (G.R.-H.); ruilope@icloud.com (L.M.R.); 6 Bioinformatics Unit, Genetics Department, Instituto de Investigación Sanitaria Fundación Jiménez Díaz-UAM, 28040 Madrid, Spain; 7 Cardiorenal Translational Laboratory, Institute of Research Imas12, Hospital Universitario 12 de Octubre, 28041 Madrid, Spain; hta@juliansegura.com; 8 Departamento de Fisiología, Facultad de Medicina, Universidad Autónoma de Madrid, 28029 Madrid, Spain; 9 Department of Vascular Physiopathology, Hospital Nacional de Parapléjicos, 45004 Toledo, Spain; megonzalezb@sescam.jccm.es; 10 Department of Vascular Physiopathology, Hospital Nacional de Parapléjicos, IDISCAM, 45004 Toledo, Spain; 11 First Department of Nephrology, Hippokration Hospital, Aristotle University of Thessaloniki, 54124 Thessaloniki, Greece; psarafidis11@yahoo.gr; 12 Hypertension Unit, Hospital Universitario 12 de Octubre, 28041 Madrid, Spain; 13 School of Doctoral Studies and Research, European University of Madrid, 28005 Madrid, Spain; 14 RICORS2040, IIS-Fundación Jiménez Díaz, UAM, 28040 Madrid, Spain; 15 Department of Biochemistry and Molecular Biology, Complutense University, 28040 Madrid, Spain

**Keywords:** chronic kidney disease, cardiorenal risk, cardiovascular disease, glycoproteins, N-glycosylation, proteomics, hypertension

## Abstract

Early diagnosis and treatment of chronic kidney disease (CKD) is a worldwide challenge. Subjects with albumin-to-creatinine ratio (ACR) ≥ 30 mg/g and preserved renal function are considered to be at no cardiorenal risk in clinical practice, but prospective clinical studies evidence increased risk, even at the high-normal (HN) ACR range (10–30 mg/g), supporting the need to identify other molecular indicators for early assessment of patients at higher risk. Following our previous studies, here we aim to stratify the normoalbuminuria range according to cardiorenal risk and identify the glycoproteins and N-glycosylation sites associated with kidney damage in subclinical CKD. Glycoproteins were analyzed in urine from hypertensive patients within the HN ACR range compared to control group (C; ACR < 10 mg/g) by mass spectrometry. A different cohort was analyzed for confirmation (ELISA) and sex perspective was evaluated. Patients’ follow-up for 8 years since basal urine collection revealed higher renal function decline and ACR progression for HN patients. Differential N-glycopeptides and their N -glycosylation sites were also identified, together with their pathogenicity. N-glycosylation may condition pathological protein deregulation, and a panel of 62 glycoproteins evidenced alteration in normoalbuminuric subjects within the HN range. Haptoglobin-related protein, haptoglobin, afamin, transferrin, and immunoglobulin heavy constant gamma 1 (IGHG1) and 2 (IGHG2) showed increased levels in HN patients, pointing to disturbed iron metabolism and tubular reabsorption and supporting the tubule as a target of interest in the early progression of CKD. When analyzed separately, haptoglobin, afamin, transferrin, and IGHG2 remained significant in HN, in both women and men. At the peptide level, 172 N-glycopeptides showed differential abundance in HN patients, and 26 showed high pathogenicity, 10 of them belonging to glycoproteins that do not show variation between HN and C groups. This study highlights the value of glycosylation in subjects not meeting KDIGO criteria for CKD. The identified N-glycopeptides and glycosylation sites showed novel targets, for both the early assessment of individual cardiorenal risk and for intervention aimed at anticipating CKD progression.

## 1. Introduction

Chronic kidney disease (CKD) is projected to be the fifth cause of death in less than twenty years due to the unmet needs for early diagnosis and treatment [1]. Early detection is key for effective management, but the current definition of CKD identifies only advanced stages [2]. Moderately increased albuminuria is one of the main criteria in CKD diagnosis and categorization with a cut-off value of urinary albumin-to-creatinine ratio (ACR) ≥30 mg/g. Moderately increased albuminuria is associated with higher cardiovascular morbimortality and renal disease progression at any stage of kidney function [3,4]. Subjects below this cut-off for ACR (normoalbuminuria) and with an estimated glomerular filtration rate (eGFR) > 60 mL/min/1.73 m^2^ do not meet the KDIGO criteria for CKD and are considered to be at no increased cardiorenal risk on the relevant KDIGO “heatmap” charts [5]. However, cardiovascular risk and renal function decline are associated with urinary albumin concentration further below the stablished cut-off for risk, i.e., within the normoalbuminuria range [6,7,8]. Cardiovascular events, increased incidence of CKD, and faster decline of eGFR have been shown in subjects with ACR values in the high-normal range (ACR = 10–30 mg/g) [6,9,10,11,12]. These clinical evidences point to subclinical renal and cardiovascular damage responsible for a negative prognosis of high-normal patients, for whom no therapeutic action is envisaged in the absence of comorbidities, supporting the “blind-spot” concept in CKD diagnosis, which is when kidney and cardiovascular injury is present but undetectable by current diagnostic criteria so that no intervention is made before damage occurs [2].

For early intervention to delay albuminuria progression and prevent a negative cardiorenal prognosis, normoalbuminuric patients at higher risk need to be identified, guided by more than the ACR, for which novel tools complementing existing risk algorithms are required. Previous studies in diabetic patients showed circulating WFDC2 and MMP-7 proteins associating with higher risk of renal decline, even within the normoalbuminuria range [13]. A classifier composed of 276 urinary peptides showed the capacity to identify early renal risk by predicting albuminuria progression also in normoalbuminuric diabetic patients [14]. We have previously shown alterations in the urinary proteome and metabolome in hypertensive subjects developing moderately increased albuminuria [15,16]. More recently, we have demonstrated how urinary molecular profiling may help to identify subclinical organ damage in patients not considered at risk. In particular, we have evidenced a metabolic signature pointing to impaired β-oxidation of fatty acids, PPAR activation, and energy metabolism dysregulation, evidencing tubular damage already in normoalbuminuric subjects [17]. Those findings were supported by a protein profile also identified in high-normal patients [18]. Interestingly, a high number of the altered proteins identified were glycoproteins.

Glycosylation is one of the most prominent and abundant posttranslational modifications, and glycoproteins participate in key biological processes such as cell attachment, signal transduction, and protein folding [19]. The analysis of the glycoproteome has revealed alterations in the lipid metabolism and inflammatory response during the course of diabetic nephropathy, establishing a differential profile between normoalbuminuric and patients with ACR in the pathological range (ACR > 30 mg/g) [20]. Glycosylation may also condition kidney fibrosis mediated by transforming growth factor β1 and lysyl oxidase 2 secretion [21] and influence LDL and HDL metabolism and lipoprotein receptor functionality [22], all together evidencing its role in kidney pathology and vascular disease, which deserve further study.

Here we aim to investigate whether glycoproteome allows stratifying the normoalbuminuria range according to cardiorenal risk and reflect molecular alterations associated with kidney damage in people currently identified as not having CKD. For such a purpose, we have identified differences in the urinary abundance of glycoproteins, both globally and from the sex perspective. The pathogenicity associated with N-glycosylation positions within the peptide sequence was also investigated to identify specific checkpoints subjective of action.

## 2. Results

**Figure 1:**
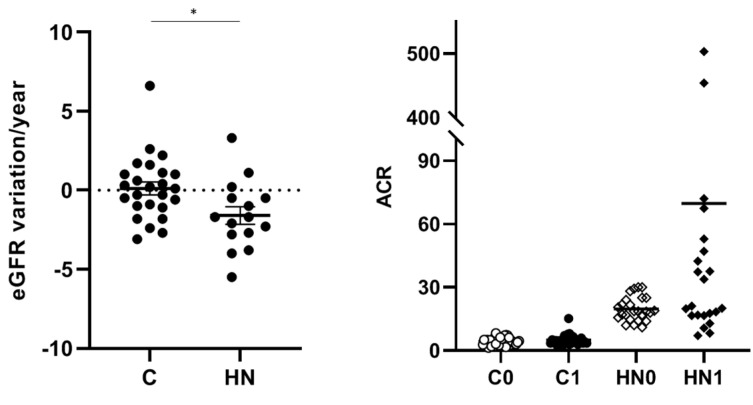
The renal function decline and ACR progression were evaluated for 8-year follow-up. eGFR variation/year was calculated with available data from annual patient check-ups for 8 years after urine collection. ACR average values were also represented by comparing basal data (C0 and HN0) with progression data after 8-year follow-up (C1 and HN1). * *p* value = 0.0156.

Renal function decline was monitored for the 8-year patient follow-up since urine sample collection. eGFR variation per year and ACR progression are shown in Figure 1 for C and HN patients. Significantly higher renal function decline was observed for HN patients compared to C patients (*p* = 0.0156). Average ACR values over time also reflect a worse evolution towards moderately high albuminuria (microalbuminuria) in HN patients compared to C patients. These data combined evidence that the HN patients included in this study actually have a worse renal evolution.

In the search for molecular and subclinical indicators of early CKD, we have firstly identified differential glycoproteins in basal urine. Secondly, we have evaluated differences at the N-glycopeptide level, paying special attention to those N-glycopeptides whose protein of origin does not show abundance variation. In this way, specific changes in these N-glycopeptides may indicate a pathological state that would otherwise go unnoticed. 

### 2.1. The Urinary Glycoproteome Differentiates Patients with Higher Cardiorenal Risk in Women and Men Not Meeting Criteria for CKD

**Figure 2:**
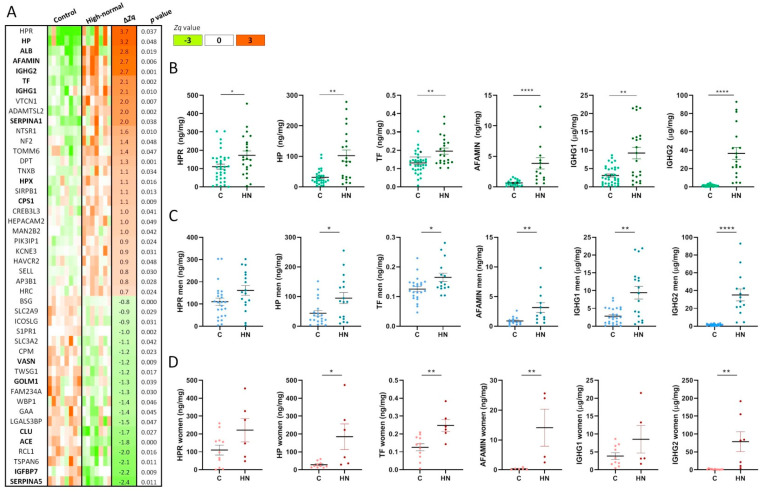
The urinary glycoproteome differentiates normoalbuminuric patients with higher cardiorenal risk. (**A**) Heat map showing differentially abundant glycoproteins in high-normal (HN) compared to control (C) patients. Significantly increased or decreased glycoproteins are represented in orange or green, respectively. A sub-set of glycoproteins were identified here, with significant alteration and a similar trend as in our previous study of the whole urinary proteome (highlighted in bold). (**B**) Protein-to-creatinine ratio differential abundance confirmed by ELISA. (**C**) Protein-to-creatinine ratio differential abundance in men. (**D**) Protein-to-creatinine ratio differential abundance in women. * *p* value < 0.05, ** *p* value < 0.01, **** *p* value < 0.0001.

The clinical data of patients are shown in Table 1. With the exception of age, no significant differences were observed in anthropometric, metabolic, and blood pressure parameters between patients stratified according to their ACR in control (C, ACR < 10 mg/g) or high-normal albuminuria (HN, ACR = 10–30 mg/g) in either of the studied cohorts (discovery and confirmation). The untargeted analysis of the glycoproteome revealed 476 N-glycosylated proteins, among which 62 showed differential abundance in the HN group compared to the C group (Supplementary Table S1). A subset of 46 N-glycoproteins were selected based on the difference between means (∆*Zq*) (Figure 2). Maximum increase in abundance corresponds to HPR (∆*Zq* = 3.7), HP (∆*Zq* = 3.2), albumin (positive control) (∆*Zq* = 2.8), AFAMIN (∆*Zq* = 2.7), IGHG2 (∆*Zq* = 2.7), TF (∆*Zq* = 2.1), and IGHG1 (∆*Zq* = 2.1) (Figure 2A). A sub-set of glycoproteins were identified here, with significant alteration and similar trend as in our previous study of the whole urinary proteome (highlighted in bold in Figure 2A), confirming the early alteration of this protein panel [18].

Glycoproteins variation in their urine concentration was further confirmed by ELISA for HPR, HP, AFAMIN, TF, IGHG1, and IGHG2 (Figure 2B). Positive correlation with age was found significant only for HPR, AFAMIN, and IGHG1 (Supplementary Table S2). AFAMIN and IGHG1 variation in HN compared to C was independent of age (*p* value = 0.004 in the age-adjusted model). None of the six proteins significantly correlated with eGFR (Supplementary Table S2).

Sex perspective was evaluated by quantifying the urinary glycoproteins levels in men and women separately in the confirmation cohort. All of them but HPR and IGHG1 remained significantly increased in HN patients, either in men (Figure 2C) or women (Figure 2D). IGHG1 preserved its significance only in men.

### 2.2. Pathogenic N-Glycopeptides Differentiate Normoalbuminuric Patients with Higher Cardiorenal Risk

A total of 1189 N-glycopeptides were identified, and their differential abundance between clinical groups was evaluated. Among them, 172 N-glycopeptides showed differential abundance in HN patients (Supplementary Table S3). Out of the 172 N-glycopeptides, 97 belong to proteins that have not shown significant alteration in abundance in HN patients, highlighting the value of the N-glycopeptidome itself in the search for new targets of action.

The N-glycosylation and the position within the protein sequence where it occurs determine protein structure and stability and may have an impact on protein adhesion properties, its potential to be secreted, and its signaling capacity, among other characteristics that ultimately define the protein function. Polyphen2 is a widely used pathogenicity predictor for aminoacid changes. We used the Polyphen2 score over five random mutations (PSaverage) on N-glycosylation sites as a proxy to determine the biological relevance of the N-glycosylation sites [23]. Supplementary Table S4 compiles the PSaverage associated with each N-glycosylation site calculated for all experimental N-glycopeptides identified, showing differential abundance in HN patients compared to C patients. Among those, Table 2 compiles the 26 N-glycopeptides that significantly varied in abundance in HN patients and with an associated PSaverage > 0.908. Ten N-glycopeptides belong to glycoproteins that do not show variation between the HN and C groups. Increased N-glycopeptides belong to CDH13, LRG1, and PCDH1 glycoproteins; decreased N-glycopeptides belong to ACAN, CD276, CNTN1, COL6A1, LSAMP, OLFM4, and SCARB2 glycoproteins. Sixteen N-glycopeptides belong to glycoproteins that also show variation in HN patients.

## 3. Discussion

The glycoproteins and N-glycopeptides here identified support our previous findings in patients with moderately increased albuminuria, and further evidence a molecular deregulation in the subclinical stage of normoalbuminuria for those individuals at higher cardiorenal risk (ACR = 10–30 mg/g). This molecular deregulation exists despite no differences in the patients’ lipid profile, GFR, or the existence of diabetes.

**Figure 3:**
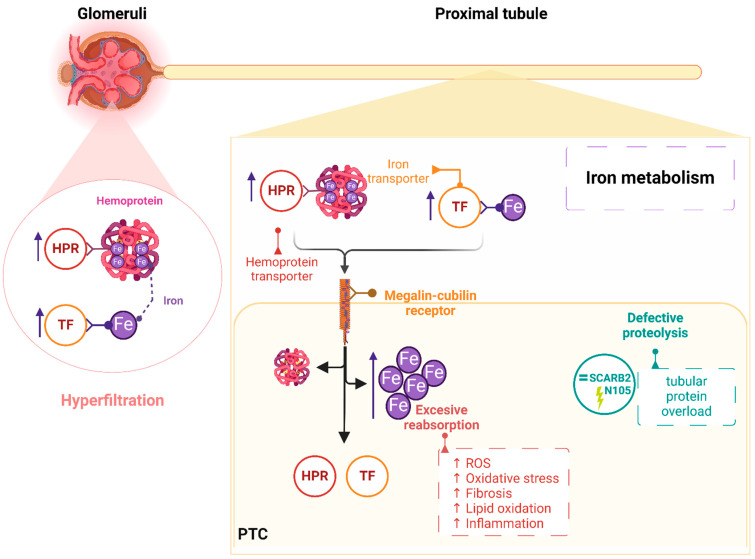
Alterations identified in the urinary glycoproteome evidence iron metabolism deregulation in the renal tubule, involving hemoprotein transporter haptoglobin related protein (HPR) and iron transporter protein transferrin (TF). The study of N-glycosylation sites within the peptide sequence revealed specific alterations of the N-glycopeptides associated with high pathogenicity, independently of unaltered levels of their glycoprotein of origin. That was the case for SCARB2 and its altered N-glycopeptide containing N105 position, pointing to the defective proteolysis of reabsorbed proteins and protein overload. Created with BioRender.com (accessed on 10 May 2024).

Albuminuria and eGFR decline have previously been associated with the glycosylation of acute-phase proteins α1-acid glycoprotein, HP, α1-antitrypsin (SERPINA1), α1-antichymotrypsin, and TF, suggesting a role for abnormal glycosylation in the underlying mechanisms of CKD [24]. The increased levels observed here for HP, SERPINA1, and TF in HN patients supports those findings and even evidence them already in an early stage. HP and AFAMIN anticipate early renal function decline in diabetic patients who have not developed albuminuria [25,26]. For HP, the authors suggest increased expression in response to tubular injury or HP leakage into the tubules, pointing to defective tubular reabsorption. Previous studies from our group in normoalbuminuric patients revealed molecular alterations in urine and extracellular vesicles, in agreement with defective tubular reabsorption [17,27]. On the other hand, HP is affected by genetic polymorphisms with two main forms (Hp1 and Hp2) showing different frequencies in the Spanish population, antioxidant capacity, and increased risk of subclinical vascular disease in diabetic subjects [28]. A different association was also observed with renal function decline, but no significant association was found between the HP genotype and albumin excretion [29]. In this study, all haptoglobin types naturally present in patients’ samples were analyzed and, considering that our patients’ cohort are within the normoalbuminuria range, we may not expect a direct influence of polymorphisms. Our results also evidence alterations in hemoprotein levels, pointing to iron metabolism deregulation (Figure 3). In conditions of glomerular filtration barrier dysfunction, a pathological filtrate of hemoproteins and reabsorption by megalin–cubilin receptors on proximal tubular cells may end in iron dissociation, hydroxyl radicals’ generation, and consequent oxidative injury [30]. In this vein, tubular iron deposition is a known phenomenon in CKD, and increased levels of iron and iron-related proteins have been found in urine in association with renal damage [31]. In this work, several iron-related proteins have been identified with increased urinary levels in HN patients, including TF, HPR, and hemopexin, pointing to a disturbed iron metabolism starting early in CKD progression [32] and further supporting the tubule as a target of interest. 

This study evidences that normoalbuminuric patients at the 10–30 mg/g ACR range have altered levels of urinary glycoproteins. Beyond that, the pathological deregulation of a protein can also be conditioned by post-translational modifications, and this is particularly relevant when there is no alteration in the concentration of the protein of origin [33,34,35,36]. That is the case for instances of the structural features of IgA N-glycosylation that have been associated with glomerular function and IgA nephropathy (IgAN), showing N-glycopeptides as better predictors of IgAN and renal function than galactose-deficient IgA1 levels measured by lectin-based ELISA [37]. Aggrecan core protein (ACAN) is a major component of the extracellular matrix, and its urine N-glycopeptide glycosylated at site N387 has here been found to be significantly varied in HN patients, despite comparable levels of ACAN with control patients. Similarly, ACAN has not shown significant abundance variation in patients with a congenital disorder of deglycosylation, but the most upregulated N-glycopeptide in fibroblasts is coincident with the one here observed, confirming a potential pathogenic role [38]. In agreement with tubular dysfunction in early CKD, the defective proteolysis of reabsorbed proteins in the proximal tubule has been suggested as a novel mechanism of tubular proteinuria in mice deficient in lysosome membrane protein 2 (LIMP-2; the murine homologue of SCARB2). No differences in megalin–cubilin expression (reabsorption) or in lysosomal abundance were observed compared to wild type, suggesting an inability to fuse lysosomes with endosomes for reabsorbed proteins degradation [39]. Ten sites were identified for human SCARB2, including the N105 here observed, which is highly conserved among vertebrates and so apparently essential for SCARB2 function [40]. In our study, no significant variation was found in SCARB2 urinary levels, but modifications in N-glycopeptide abundance glycosylated at N105 may appear to already condition SCARB2 function within the normoalbuminuric range in what refers to tubular protein overload (Figure 3). The proteins here identified as CD276, OLFM4, and PCDH1 are expressed mostly in the tubule and minimally in the glomerulus [41] and showed unaltered levels in urine but alteration in specific N-glycopeptides.

Overall, this study covers the analysis of the urinary proteins further than their abundance levels. The identified N-glycopeptides and their glycosylation sites may play a key role in the pathological development of albuminuria and associated cardiorenal risk, opening new research lines and potential ways of action so far undiscovered. In agreement with our previous studies, tubular reabsorption is also raised here as a course of action, which needs to be investigated in greater depth in approaching early CKD.

As a limitation, peptide deamidation could be both a biological process and an artefact. Here, we manually examined every deamidated residue, with the limitation that some potentially intriguing glycosylated peptides were missed in the analysis. Related to the biological role of N-glycopeptides, we have investigated changes in the urine concentration of glycoproteins and their N-glycopeptides, together with the pathogenicity associated to their specific N-glycosylation sites. The study of the N-glycosylation pattern itself (N-glycans) was out of the scope of this work. The N-glycosylation positions here shown with high pathogenicity may be further investigated to decipher their specific role in CKD and precisely define their value as therapeutic targets. Monitoring peptide concentrations during follow-up could also provide a better understanding of glycoprotein association with CKD. For sample size limitations, the influence of renoprotective drugs on N-glycopeptides and glycosylation pattern could not be properly accomplished, but it is another field of interest that deserves future investigation.

In conclusion, this study supports the need to molecularly stratify the normoalbuminuria condition for precise assessment of individual cardiorenal risk. We present a urinary glycoprotein panel able to differentiate patients with cardiorenal risk but not meeting criteria for CKD. The sentinel signatures here identified may help to expand current tools in daily clinical practice. Cardiorenal risk stratification may benefit from the discovery of these glycoproteins in patients with unaltered lipid profiles or GFR, with the ultimate goal of understanding the pathological ways of action and target early intervention to turn the tide on the increasing global burden of CKD and its associated adverse cardiovascular outcomes. The data shown here also support the need to go beyond alterations in protein abundance by analyzing posttranslational modifications such as glycosylation. The existence of N-glycosylation sites whose removal reveal pathogenic properties suggests potential targets to monitor or where to intervene, so that new possibilities arise in therapeutics engineering for managing kidney disease [42].

## 4. Materials and Methods

### 4.1. Patients’ Selection and Urine Samples Collection

Hypertensive subjects under chronic renin-angiotensin system (RAS) suppression, with eGFR > 60 mL/min/1.73 m^2^ and ACR values within the normoalbuminuria range (< 30 mg/g) were recruited from the Hypertension Unit in Hospital 12 de Octubre (Madrid) and classified as the control group (C) if ACR < 10 mg/g or the high-normal group (HN) if ACR = 10–30 mg/g. Patients between 40 and 75 years were included. Subjects with secondary hypertension were excluded. Blood pressure was measured, according to recommendations, as the mean of three readings measured at the office with a validated semiautomatic oscillometer device, after 5 min resting in a sitting position [43]. Urine samples were collected in sterile recipients, centrifuged at 3000× *g* for 10 min and stored at –80ºC until analysis [18]. 

### 4.2. Glycoproteins Quantitation by Untargeted Mass Spectrometry

#### 4.2.1. Protein Digestion and Peptide Isobaric Labelling

Proteins were digested with modified trypsin (Sequencing Grade Modified Trypsin; Promega, Maddison, WI, USA) using a protein:trypsin ratio of 30:1 (*w*/*w*) and using the FASP (Filter Aided Sample Prep, Pall, New York, NY, USA) protocol as previously described [44]. The resulting peptides were desalted onto Oasis-HLB cartridges (Waters, Milford, MA, USA) and dried down. For stable isobaric labelling, the peptides were dissolved in 100 mmol/L triethylammonium bicarbonate buffer, and the peptide concentration was determined by measuring amide bonds with the Direct Detect system (Millipore, Merck, Rahway, NJ, USA). Equal amounts of total peptides per sample were labelled using 8-plex iTRAQ^®^ reagents (isobaric tags for relative and absolute quantitation, AB Sciex, Nieuwerkerk aan den IJssel, The Netherlands) according to manufacturer’s protocol and combined. 

#### 4.2.2. N-Glycopeptides Enrichment for LC-MS/MS Analysis

To quantify the glycoproteins, tryptic N-glycopeptides were isolated by a FASP-based N-linked glycopeptide capture method (N-Glyco-FASP), adapted from a previously published protocol [45]. Briefly, 40 µL of H₂O:binding buffer (40 mM Tris (ACS Reagent Grade; Cytiva, Marlborough, MA, USA)/HCl (Hydrochloric acid fuming 37%; Merck, Rahway, NJ, USA), 2 mM MnCl₂ (ACS Reagent Grade; Merck), 2 mM CaCl₂ (>95%; Panreac), 1 M NaCl₂ (ACS Reagent grade > 99.5%; Sigma-Aldrich, St. Louis, MO, USA)) (1:1), and 100 µL of 40 mM ammonium bicarbonate (>99%; Sigma-Aldrich) were added to the labelled peptides, which were then loaded into 10 kDa centrifugal filter devices (NanoSep 10k Omega, Pall Life Sciences, New York, NY, USA) and incubated on a filter with wheat germ agglutinin (WGA) lectin (6 µg/µL, L9640, Sigma-Aldrich) for 1 h. Non-glycosylated peptides were washed away and discarded. The retained N-glycopeptides were incubated with PNGase F (3U, Sigma-Aldrich) for 3 h at 37 °C. PNGase F enzyme specifically cuts the bond between the asparagine (Asn) residue and the glycidic part of the glycopeptide, deamidating Asn to aspartic acid (Asp). Peptides were desalted on C18 OmiX columns (Agilent Technologies, Santa Clara, CA, USA) and concentrated. To identify and discard previous deamidations occurring naturally, one part is injected before and one part after purification, and Asp and Gln deamidations in the latter that are not detected in the former are considered as the actual glycosylations.

#### 4.2.3. Enriched N-Glycopeptides Analysis by LC-MS/MS

Labelled N-glycopeptides were analyzed by LC-MS/MS using a C18 reversed-phase nanocolumn (75 μm ID × 50 cm, 2 μm particle size, Acclaim PepMap RSLC, 100 C18; ThermoFisher Scientific) in a continuous acetonitrile (hypergrade for LC-MS LiChrosolv; Sigma-Aldrich) gradient consisting of 5–32% B for 240 min and 32–90% B for 5 min (B = 100% acetonitrile, 0.1% formic acid) at 200 nL/min on an Ultimate 3000 HPLC system (Thermo Scientific, San Jose, CA, USA) coupled to an Orbitrap Fusion mass spectrometer (Thermo Scientific). An enhanced Fourier-transform resolution spectrum (full width at half-maximum (FWHM) resolution of 70,000 at m/z 200) followed by the MS/MS spectra from the most intense parent ions were obtained along the chromatographic run. Dynamic exclusion was set at 40 s. The resulting spectra were analyzed with Proteome Discoverer (2.1.0.81 version, Thermo Fisher Scientific) and SEQUEST-HT (Thermo Fisher Scientific). The Uniprot database was used for protein identification, containing all sequences from humans and contaminants (14 May 2016, 70611 entries). The contaminants database was downloaded from MaxQuant and includes common contaminants from the “common Repository of adventitious Proteins (cRAP)” database. Carbamidomethylcysteine and iTRAQ modifications at the N-terminal and Lys’s residues were defined as fixed modifications, and methionine oxidation and Asn deamidation were selected as dynamic modifications. Peptide identification was carried out using the probability ratio method [46], and the false discovery rate (FDR) was calculated using inverted databases and the refined method [47], with an additional filtering for a precursor mass tolerance of 15 ppm [48]. Identified peptides had an FDR ≤ 1%.

#### 4.2.4. Differential Quantitation between Clinical Groups

Differential quantitation was performed at two levels: glycoproteins and N-glycopeptides. For quantitative analysis, the SanXoT program [49] integrates reporter intensities from the spectrum level to the peptide level, and subsequently to the protein level, to quantify the relative abundance of each protein according to the WSPP (weighted spectrum, peptide, and protein) statistical model, as previously described [50]. In this model, changes are quantified as log2-ratios and expressed as standardized variables, specifically Z-scores for peptides (Zp) and proteins (Zq), which represent the normalized values in units of standard deviation (SD) according to their respective estimated variances. Glycoprotein and N-glycopeptide abundance changes between groups were calculated by comparing their *Zq* and *Zp* mean values, respectively (C, *n* = 8; HN, *n* = 7). Significant variation was considered if *p* value < 0.05.

### 4.3. Targeted Glycoproteins Quantitation and Sex Differences

Haptoglobin-related protein (HPR), haptoglobin (HP), transferrin (TF), afamin (AFAMIN), immunoglobulin heavy constant gamma 1 (IGHG1), and immunoglobulin heavy constant gamma 2 (IHGH2) were quantified by ELISA in a different patients’ cohort, and protein concentration was expressed as normalized by urinary creatinine. Data were additionally analyzed separately for women and men. Manufacturer protocols were followed, and optimal conditions were established for urine samples (Supplementary Table S5). GraphPad Prism 8 software (version 8.0.2) was used for statistical analysis. The ROUT (robust regression and outlier removal) method was applied based on the FDR (95% confidence level), setting the Q value to 5%. The Shapiro–Wilk normality test was performed for the subsequent application of the *t*-test or Mann–Whitney test. Differences were considered significant if *p* < 0.05. Spearman correlation was calculated for age and eGFR by GraphPad Prism 8 software (version 8.0.2). The differences found in glycoprotein abundance were adjusted for age if significant correlation was found, using logistic regressions performed with RStudio software (version 2022.02.0 + 443). The renal function decline and ACR progression were evaluated for patients included in the confirmation cohort (n = 63) for 8-year follow-up since urine sample collection. eGFR variation/year and ACR average values were calculated with available data from annual patient check-ups. A patient was included if data from a minimum of 4 revisions were available.

### 4.4. Pathogenicity Associated to Differential N-Glycosylation Sites

**Figure 4:**
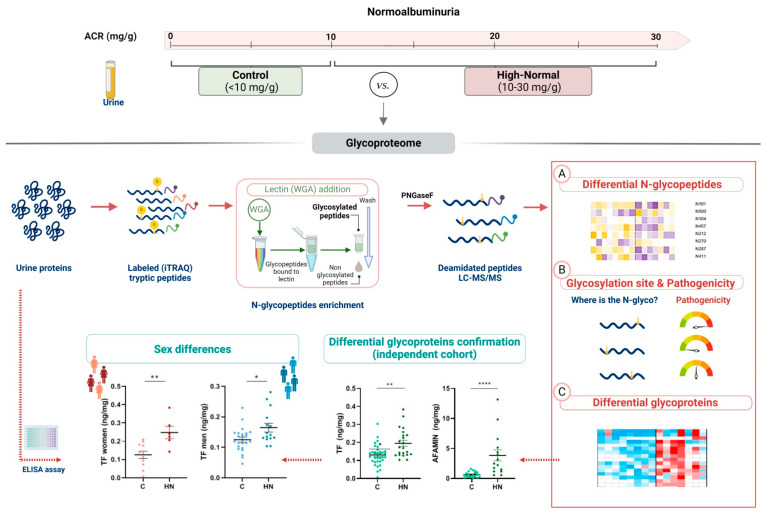
Schematic view of the study workflow. Urinary proteins from control (C) and high-normal (HN) groups were digested and labeled for differential quantitation (iTRAQ). The N-glycopeptides were enriched and analyzed by untargeted LC-MS/MS (discovery phase). Glycoproteins showing altered abundance in HN patients were identified and confirmed by ELISA assays. Sex perspective was evaluated. At the peptide level, differential N-glycopeptides were also identified, irrespective of the abundance variation of their glycoprotein of origin. N-glycosylation sites were identified, and their associated pathogenicities were quantified. Created with BioRender.com. * *p* value < 0.05, ** *p* value < 0.01, **** *p* value < 0.0001.

The pathogenicity associated with the identified N-glycosylation sites was additionally investigated. A schematic view of the multi-analysis workflow is shown in Figure 4. N-glycopeptides with differential abundance between C and HN groups were identified, and the pathogenicity associated with their N-glycosylation sites was calculated. N-glycosylations were mapped into human protein sequences obtained from eggNOG v4.5 [51]. N-glycosylations mapping the same site were merged to remove redundancy. In order to predict the phenotypic consequences of preventing glycosylation, we used a computational (in-silico) approach. For each glycosylation site we do the following: (1) take the protein sequence and introduce an amino acid change in the glycosylation site that mimics a missense genomic variation, swapping the asparagine for a random amino acid; (2) measure the pathogenicity of the change using Polyphen2 [52] provided as the Polyphen score (PS); (3) steps 1 and 2 are performed five times, obtaining 5 PS; (4) we define the PSaverage score as the average of the PS obtained for the amino acid changes for each glycosylation site. When the pathogenic value is >0.908, the associated pathogenicity prediction is “probably damaging”, thus we chose 0.908 as the cut-off value to identify the highly-pathogenic N-glycopeptides differentially expressed between C and HN subjects.

## Figures and Tables

**Table 1:** Baseline clinical data of hypertensive patients under chronic RAS suppression classified in control group (ACR < 10 mg/g) and high-normal group (ACR = 10–30 mg/g). Values are expressed as number (%) for categorical variables and mean ± standard deviation for continuous variables.

	Untargeted Mass Spectrometry (LC-MS/MS) (Discovery Cohort)			Targeted Quantitation (ELISA) (Confirmation Cohort)		
	Control (C) (*n* = 8)	High-Normal (HN) (*n* = 7)	*p* Value	Control (C) (*n* = 38)	High-Normal (HN) (*n* = 25)	*p* Value
Age (years)	55 ± 8	63 ± 6	0.0535	58 ± 7	63 ± 6	0.0140
Sex (% male)	8 (100)	7 (100)	>0.9999	25 (66)	18 (72)	0.7829
Cholesterol total (mg/dL)	161 ± 37	154 ± 34	>0.9999	180 ± 32	171 ± 29	0.4158
Triglycerides (mg/dL)	104 ± 34	113 ± 44	0.6733	109 ± 39	118 ± 50	0.7754
Cholesterol HDL (mg/dL)	44 ± 8	48 ± 15	0.6794	54 ± 15	54 ± 16	0.7500
Cholesterol LDL (mg/dL)	95 ± 35	84 ± 30	0.8065	103 ± 28	93 ± 30	0.3628
Glycemia (mg/dL)	104 ± 9	103 ± 20	0.3792	103 ± 11	104 ± 17	0.7610
Uric acid (mg/dL)	6 ± 1	7 ± 2	0.2426	6 ± 1	6 ± 1	0.0925
ACR (mg/g)	4 ± 2	18 ± 4	0.0003	4 ± 2	20 ± 6	<0.0001
Diabetes mellitus type 2 (%)	25	14	>0.9999	11	16	0.7019
SBP (mmHg)	136 ± 15	142 ± 13	0.2645	139 ± 15	141 ± 13	0.6883
DBP (mmHg)	85 ± 8	82 ± 7	0.7167	83 ± 8	82 ± 8	0.3767
**Antihypertensive Treatment (%)**						
iECAs	38	29	>0.9999	24	20	0.7683
ARA	50	57	>0.9999	68	64	0.7965
Diuretic	50	43	>0.9999	34	52	0.3007
Calcium channel blocker	25	71	0.1319	42	76	0.0053
α-blocker	50	0	0.0769	26	0	0.1474
β-blocker	38	29	>0.9999	13	36	0.5755
**Other Treatment (%)**						
Anticoagulant	13	0	>0.9999	5	4	>0.9999
Lipid lowering	50	71	0.6084	63	56	0.5965
Antidiabetic	25	14	>0.9999	8	8	>0.9999

**Table 2:** Experimental N-glycopeptides showing differential abundance in patients with early cardiorenal risk and N-glycosylation sites with high pathogenicity (>0.908). The protein, peptide sequence, and N-glycosylation sites with associated pathogenicity are shown, together with variation trends of the N-glycopeptide and glycoprotein of origin. **N$**: glycosylation site. Increased peptides are represented in yellow and decreased peptides in purple (Supplementary Table S3).

Peptide Sequence Identified by MS/MS	Gene	*Zp* Values (Peptide Abundance) C - HN																	N-Glycosylation Site	Pathogenicity Score	HN vs. C(Peptide)	HN vs. C(Protein)
***N-glycopeptidome specific signature***																						
**N$**ITEGEAR	*ACAN*																		N387	0.9158	↓	-
QEDLSVGSVLLTV**N$**ATDPDSLQHQTIR	*CDH13*																		N500	0.9970	↑	-
TALFPDLLAQG**N$**ASLR	*CD276*																		N104	0.9813	↓	-
GTEWLV**N$**SSR	*CNTN1*																		N457	0.9998	↓	-
**N$**FTAADWGQSR	*COL6A1*																		N212	0.9968	↓	-
QLDM_LDLSN**N$**SLASVPEGLWASLGQPNWDM_R	*LRG1*																		N270	0.9990	↑	-
STEGQSSLTVTNVTEEHYG**N$**YTC#VAANK@	*LSAMP*																		N287	0.9984	↓	-
L**N$**DTTLQVLNTWYTK@	*OLFM4*																		N411	0.9935	↓	-
YGTALVHLYV**N$**ETLA**N$**R	*PCDH1*																		N813/N818	0.9998/0.9999	↑	-
ANIQFGD**N$**GTTISAVSNK@	*SCARB2*																		N105	0.9077	↓	-
***Other N-glycopeptides***																						
ELPGVC#**N$**ETMMALWEEC#K@PC#LK@	*CLU*																		N103	0.9996	↓	↓
ML**N$**TSSLLEQL**N$**EQFNWVSR	*CLU*																		N363	0.9242	↓	↓
AVLVN**N$**ITTGER	*GOLM1*																		N109	0.9983	↓	↓
LF**N$**VTPQDEQK@	*ICOSLG*																		N102	0.9674	↓	↓
TDNSLLDQALQ**N$**DTVFLNMR	*ICOSLG*																		N186	0.9896	↓	↓
TD**N$**SLLDQALQ**N$**DTVFLNMR	*ICOSLG*																		N186	0.9896	↓	↓
TDNSLLDQALQ**N$**DTVFLNM_R	*ICOSLG*																		N186	0.9896	↓	↓
EEQY**N$**STYR	*IGHG1*																		N180	0.9551	↑	↑
TK@PREEQF**N$**STFR	*IGHG2*																		N176	0.9916	↑	↑
EEQF**N$**STFR	*IGHG2*																		N176	0.9916	↑	↑
AL**N$**ATLHS**N$**LLC#RPGPGLGPDNQTEER	*KCNE3*																		N22	0.9872	↑	↑
AQAGLEEALLAPGFG**N$**ASG**N$**ASER	*NTSR1*																		N37	0.9399	↑	↑
VVGVPYQG**N$**ATALFILPSEGK@	*SERPINA5*																		N262	0.9475	↓	↓
AFY**N$**ESWER	*SLC2A9*																		N90	0.9788	↓	↓
**N$**LSDIDLM_APQPGV	*TOMM6*																		N61	0.9951	↑	↑
LHEIT**N$**ETFR	*VASN*																		N117	0.9184	↓	↓

## Data Availability

The mass spectrometry proteomics data have been deposited at the ProteomeXchange Consortium via the PRIDE partner repository with the dataset identifier PXD049688.

## Supplementary Materials

The following supporting information can be downloaded at: https://www.mdpi.com/article/10.3390/ijms25137005/s1.
