# Supplementary material for: The Urinary Glycopeptide Profile Differentiates Early Cardiorenal Risk in Subjects Not Meeting Criteria for Chronic Kidney Disease
Source: Int J Mol Sci. 2024 Jun 26;25(13):7005. doi: 10.3390/ijms25137005 (PMC11241500; doi:10.3390/ijms25137005)
Supplement: Supplementary file 1 [file ijms-25-07005-s001.zip › SupplTable3correlationsAgeGFR.pdf]

Supplemental Table S3. Proteins correlation with age and eGFR.

	HPR	HP	TF	AFAMIN	IGHG1	IGHG2
**Age**						
p value	0.045	0.459	0.277	0.041	0.019	0.063
Spearman r	0.262	0.099	0.145	0.313	0.305	0.248
**eGFR**						
p value	0.490	0.548	0.901	0.604	0.370	0.982
Spearman r	0.093	-0.082	0.017	-0.082	-0.121	-0.003
